# Effects of Ankaferd Hemostat on red blood cell aggregation: a hemorheological study

**DOI:** 10.3906/sag-1808-60

**Published:** 2019-02-11

**Authors:** Rafiye ÇİFTÇİLER, Salih AKSU, Neslihan DİKMENOĞLU FALKMARKEN, Ibrahim Celalettin HAZNEDAROĞLU

**Affiliations:** 1 Department of Hematology, Faculty of Medicine, Hacettepe University, Ankara Turkey; 2 Department of Physiology, Faculty of Medicine, Hacettepe University, Ankara Turkey

**Keywords:** Ankaferd Hemostat, erythrocyte aggregation, hemorheology

## Abstract

**Background/aim:**

Ankaferd hemostat (ABS; Ankaferd blood stopper, İstanbul, Turkey) is a prohemostatic agent affecting erythrocytes. The hemostatic action of ABS depends upon fibrinogen gamma chain, prothrombin, and red blood cells. The aim of this study was to assess the effects of ABS on erythrocyte aggregation via hemorheological analyses.

**Materials and methods:**

To measure erythrocyte aggregation, blood samples were obtained from healthy, nonsmoker volunteers who had not taken any medication in the previous 10 days. One mL of blood was placed into the laser-assisted optical rotational cell analyzer (LORCA), into the chamber formed by the gap between two concentric glass cylinders. The solution prepared with ABS and saline was added to blood in incremental amounts of 10 µL, 20 µL, 30 µL, 40 µL, 50 µL, 60 µL, 70 µL, and 100 µL. Erythrocyte aggregation was determined by laser-assisted optical rotational cell analyzer at 37 °C.

**Results:**

AMP was found to be 17.7 ± 2.1 au in the blood without ABS, whereas it was lower in the blood with ABS. AMP was 16.0 ± 3.3 in the ABS-added blood group. RBC aggregates did not form faster when cells contacted ABS. The t t½ value was 4.6 ± 2.6 in the ABS-added blood group and 1.9 ± 0.20 in the control group. Aggregation was faster in the control group (P = 0.03). AI, which is a combination of AMP and t½, was lowered in the ABS group (48.7 ± 12.3) compared to the control group (65.8 ± 1.6) (P = 0.02). It was notable that the γIsc max (sec-1) value of the control was higher (200 ± 106) than the ABS-added blood group (141 ± 51.0).

**Conclusion:**

ABS has antierythroid aggregation effect. ABS inhibits pathological aggregation of red blood cells. Antithrombotic clinical effects of ABS may be ascribed to the antierythroid aggregan actions of the drug.

## 1. Introduction

Ankaferd hemostat (ABS; Ankaferd blood stopper, İstanbul, Turkey) is a hemostatic agent affecting red blood cell (RBC) physiology (1). Ankaferd has been used as a hemostatic agent for the management of the distinct types of clinical bleedings (2). ABS is effective on cellular hemostasis. The hemostatic effect of ABS depends upon the quick promotion of a protein network, particularly fibrinogen gamma, in relation to the erythrocyte aggregation (1). ABS has been tested in numerous clinical trials. Thrombosis associated with ABS has not been observed in any clinical study. On the contrary, antiplatelet and antithrombin effects of ABS have been demonstrated (3). The effects of ABS on RBC aggregation have not been previously investigated. 

The viscosity of the blood is a measure of its inner resistance to flow. It is an important factor in the cardiovascular system circulation (4,5). Erythrocyte deformability, erythrocyte aggregation, plasma viscosity, and hematocrit level determine the blood viscosity. Erythrocyte deformability is the capability of RBCs to modify shape as they move through the circulation. The viscosity and resistance of the blood flow are decreased by erythrocyte deformability. This characteristic is especially significant during the transition across capillaries smaller than the diameter of erythrocytes (6). In contrast to the platelets, RBCs aggregate spontaneously. Under the normal conditions, RBC aggregates to be similar to stacks of coins. This phenomenon was called as rouleaux (7). Zijlstra, first defined the technique for the quantification of RBC aggregation procedure by measuring the decrements in the light backscatter and suggested the name ‘*syllectometry*’ (8). Their principles had been confirmed in further studies (9). The *syllectometry* principle for measuring the RBC aggregation has been used since then by many researchers and is also applied as the laser-assisted optical rotational cell analyzer (LORCA) (10). 

ABS could inhibit pathological accumulation of circulating blood cells so that vessel thrombosis may not occur in the presence of ABS-induced antihemorrhagic effects. The hemostatic action of ABS depends upon the fibrinogen gamma chain, prothrombin, and red blood cells. The aim of this study was to assess the effects of ABS on erythrocyte aggregation via hemorheological analyses. Elucidation of the interrelationships between ABS and RBC physiology is important for the enlightening of the pharmacobiological action of the drug particularly for its clinical applications.

## 2. Materials and methods 

### 2.1. Instrument description

The basic instruments were the laser, thermostated measuring system, stepper motor, and video camera. The laboratory setup consisted further of an IBM-compatible PC plus printer.

### 2.2. Sample preparation

Human blood was anticoagulated with EDTA. The solution was prepared with ABS and saline. The mixing ratio was ABS/saline = 1/10. ABS alone could not be used because the clot was formed when contacted with blood. Prior to each measurement, blood samples were oxygenated for 10–15 min to mimic the arterial blood composition. Following oxygenation, 1 mL of whole blood was placed into the chamber formed between the glass cylinders. The solution prepared with ABS and saline was added to blood in incremental amounts of 10 µL, 20 µL, 30 µL, 40 µL, 50 µL, 60 µL, 70 µL, and 100 µL. Informed consent for the procedure was obtained from blood donors for this study.

### 2.3. Erythrocyte aggregation 

Human RBC aggregation was investigated with an automatic hemorheological analyzer. Erythrocyte aggregation was determined within 1 h of the withdrawal of blood from the volunteer, since erythrocyte aggregation changes with time. Aggregation parameters were measured by LORCA at 37 °C. Following oxygenation, 1 mL of whole blood was placed into the chamber formed between the glass cylinders. The solution prepared with ABS and saline was added to blood in incremental amounts of 10 µL, 20 µL, 30 µL, 40 µL, 50 µL, 60 µL, 70 µL, 80 µL, 90 µL, and 100 µL. Aggregation indices were measured with twenty different ABS quantities and two control groups. Since whole blood does not allow the laser beam to traverse through the chambers as in erythrocyte aggregation measurement depends on backscattered light. With the aggregation of erythrocytes, the intensity of this laser light decreases, and this decrease is recorded as a syllectogram. LORCA software calculates the amplitude (AMP, representing the total extent of aggregation, measured in au unit), aggregation half-time (t½, time that passes until the peak intensity is reduced by half, reflects the kinetics of aggregation, represented in seconds), γIsc max (sec−1) (threshold shear rate needed to prevent aggregation) and aggregation index which is an index associated both AMP and t½ parameters in its formula (AI, a larger index represents greater and/or faster aggregation, shown in % as unit) and from this syllectogram (7,10,11). An increase in AMP and a decrease in t½ increase AI were observed. 

### 2.4. Statistical analysis

Statistical analyses were performed using the SPSS software version 25. The variables were investigated using visual (histograms, probability plots) and analytical methods (Kolmogorow–Simirnov/Shapiro–Wilk’s test) to determine whether or not they are normally distributed. Data were analyzed separately with Mann–Whitney U test and also with independent samples Student’s t test in order to test the validity of data with regards to parametric assumptions. 

## 3. Results

### 3.1. Effects of ABS on erythrocyte aggregation

Results ABS application lowered AMP compared to blood without ABS. AMP is an indicator of the magnitude of erythrocyte aggregation and its value was found to be 17.7 ± 2.1 au in the blood without ABS, whereas it was lower in the blood with ABS. AMP was 16.0 ± 3.3 in the ABS-added blood group. Statistical difference was not observed between the two groups (P = 0.39).

The kinetics of the aggregation process is also expressed by the half-time (t½) value. Because t½ is the time necessary to reach 50% of complete aggregation level, a lower t½ reflects a faster aggregation process. RBC aggregates do not form faster when cells contact ABS. The t½ value was 4.6 ± 2.6 in the ABS-added blood group and 1.9 ± 0.20 in the control group. Aggregation was faster in control group (P = 0.03).

AI, which is a combination of AMP and t½, is lower in the ABS group (48.7 ± 12.3) compared to that in the control group (65.8 ± 1.6) (P = 0.02). Aggregation parameters are given in Figure. γIsc max (sec−1) is threshold the shear rate needed to prevent aggregation. The measured γIsc max (sec−1) that prevented aggregation was presented in Table. It was notable that the γIsc max (sec−1) value of the control was higher (200 ± 106) than that of the ABS-added blood group (141 ± 51.0). However, it was not statistically significant (P = 0.32). Aggregation parameters are shown in Table.

**Figure 1:**
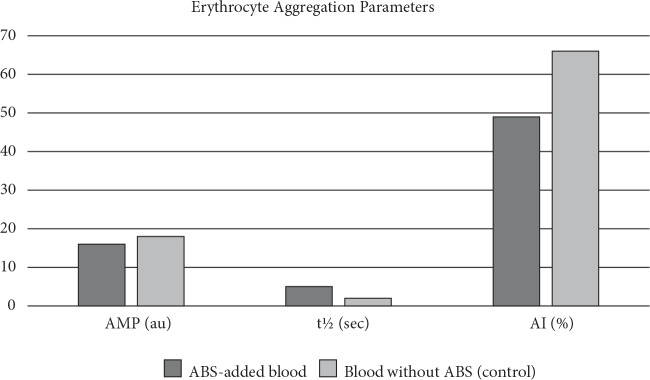
Effect of Ankaferd hemostat (ABS) on erythrocyte aggregation index (AI), amplitude (A) and aggregation half-time (t½).

**Table 1:** Effect of Ankaferd hemostat (ABS) on red blood cell (RBC) aggregation indices.

	ABS-added blood	Blood without ABS (control)	P
AMP (au)	16.0 ± 3.3	17.7 ± 2.1	0.39
t½ (sec)	4.6 ± 2.6	1.9 ± 0.20	0.03
AI (%)	48.7 ± 12.3	65.8 ± 1.6	0.02
γIsc max (sec−1)	141 ± 51.0	200 ±106	0.32

Abbreviations: AI: aggregation index; AMP: amplitude; t½: aggregation for half-time;γIsc max (sec−1): threshold shear rate needed to prevent aggregation.

## 4. Discussion

In this study, the antierythroid aggregation effects of ABS have been described. ABS inhibits pathological aggregation in red blood cells so that thrombosis may not take place in the presence of antihemorrhagic effect of ABS on clinical grounds. Previously, antiplatelet and antithrombin effects of ABS were already shown (3). The effects of ABS on RBC aggregation may be implicated in the antithrombin effects of ABS.

The hemostatic action of ABS depends upon fibrinogen gamma chain, prothrombin, and red blood cells (1). Rather than affecting an individual clotting factor, this protein network affects the entire physiological hemostatic procedure that controls bleeding (2,12). Prohemostatic and antithrombin activities of ABS were shown to be associated with fibrinogen gamma chain and prothrombin with functional proteomic analyzes. Fibrinogen gamma chain was shown to be decreased at first and then increased during the hemostatic activity of ABS in plasma and serum samples based on functional proteomic analyses. In addition to the prothrombin levels in serum that decreased first and thereafter enhanced following the generation of the ABS-induced protein network. Effects of ABS on these two molecules seem to be associated with pro-hemostatic and anti-thrombin activities of the hemostatic agent (13). Egin et al. suggested that ABS-induced antihemorrhagic effects seem to happen by the ‘balanced hemostasis’ via preventing thrombin-induced thrombotic events in the vascular endothelial space (3). The antithrombin activity of ABS is important especially in cardiac surgery. ABS was shown not to have the risk of thrombin-induced thrombotic complications by cardiac surgeons during open heart surgery (14–17). The demonstration of the antierythroid aggregation of ABS is an important result in terms of the antihemorrhagic effect that does not create a risk of thrombosis. 

Koluman et al. investigated the fate of antioxidant ingredients of ABS when exposed to synthetic gastric fluid. Furthermore, they demonstrated the safety profile for ABS focusing on the presence of pesticides, mycotoxins, heavy metals, genetically modified organisms, and dioxin. Time of Flight Mass Spectroscopy spectra showed the presence of several antioxidant molecules (such as tocotrienols, members of the vitamin E family, tryptophan, estriol, galangin, apigenin, oenin, 3,4-divanillyltetrahydrofuran, tertiary butylhydroquinone, thymol, butylated hydroxyanisole, butylated hydroxytoluene, lycopene, enoxolone/glycyrrhetinic acid or glycyrrhetic acid, and tomatine), which may have clinical implications in the pharmacological activities of ABS. They showed that the concentrations of the antioxidants and other specific molecules in the ABS were not affected after the exposure to the synthetic gastric fluid (18).

Akar et al. demonstrated the presence of Fe (III) ions and, at very low concentrations, some other trace elements inside ABS. Human fibrinogen immediately recognizes iron. Thus, iron can play an important role in the ABS related cellular hemostasis located in the junction of RBC–fibrinogen interactions (19). The antithrombotic effect of ABS may be associated with the presence of Fe (III) ions. Although with different pathogenic mechanisms, both iron deficiency and overload have been associated with an increased thrombotic risk in experimental and clinical studies (20). Future studies should focus on the association between ABS-related high iron content, fibrinogen gamma, and vital erythroid aggregation. Likewise, more studies are needed to elucidate the pharmacophysiology of the antithrombotic effect of ABS. Antiplatelet and antithrombin effects of ABS may also be related to antierythroid aggregation effects that we have found in the present study.

In conclusion, ABS has an antierythroid aggregation effect. ABS could inhibit pathological aggregation of red blood cells so that thrombosis may not occur in the presence of the antihemorrhagic effects of ABS.

## References

[ref0] (2010). Evaluation of hemostatic effects of Ankaferd as an alternative medicine. Altern Med Rev.

[ref1] (2008). Haemostatic actions of the folkloric medicinal plant extract Ankaferd Blood Stopper®. Journal of International Medical Research.

[ref2] (2015). Anti-thrombin activity of Ankaferd Hemostat in relation to the platelet functions. Turkiye Klinikleri Journal of Hematology Special Topics.

[ref3] (1998). Influence of graft material on blood rheology and plasma biochemistry following insertion of an infrainguinal bypass graft. British Journal of Surgery.

[ref4] (2009). and circulation. Clinical Hemorheology and Microcirculation.

[ref5] (2011). Effects of oral acrylamide intake on blood viscosity parameters in rats. Clinical Hemorheology and Microcirculation.

[ref6] (2001). The laser‐assisted optical rotational cell analyzer (LORCA) as red blood cell aggregometer. Clinical hemorheology and microcirculation.

[ref7] (1958). a new method for studying rouleaux formation of red blood cells. Acta physiologica et pharmacologica neerlandica.

[ref8] (1963). Quantitative evaluation of the rate of rouleaux formation of erythrocytes by measuring light reflection (“ syllectometry”). Proceedings of the Koninklijke Nederlandse Akademie van Wetenschappen Series C Biological and medical sciences.

[ref9] (1994). Laser-assisted optical rotational cell analyser (LORCA); I. a new instrument for measurement of various structural hemorheological parameters. Clinical Hemorheology and Microcirculation.

[ref10] (2009). New guidelines for hemorheological laboratory techniques. Clin Hemorheol Microcirc.

[ref11] (2010). Ultrastructural and morphological analyses of the in vitro and in vivo hemostatic effects of Ankaferd Blood Stopper. Clinical and Applied Thrombosis/Hemostasis.

[ref12] (2010). Functional proteomic analysis of Ankaferd Blood Stopper. Turkish Journal of Hematology.

[ref13] (2015). Ankaferd blood stopper decreases postoperative bleeding and number of transfusions in patients treated with clopidogrel: a double-blind, placebo-controlled, randomized clinical trial. The Heart Surgery Forum.

[ref14] (2015). Local use of ankaferd blood clotter in emergent beating heart coronary artery bypass grafting. The open cardiovascular medicine journal.

[ref15] (2010). A new practical alternative for the control of sternal bleeding during cardiac surgery: Ankaferd Blood Stopper. Heart Surgery Forum.

[ref16] (2012). Molecular basis of Ankaferd-induced hemostasis in the management of sternal bleeding. Heart Surgery Forum.

[ref17] (2016). Qualitative/chemical analyses of Ankaferd hemostat and its antioxidant content in synthetic gastric fluids. BioMed Research International.

[ref18] (2015). High iron content of Ankaferd hemostat as a clue for its hemostatic action of red blood cell origin. Blood Coagulation and Fibrinolysis.

[ref19] (2008). Iron and thrombosis. Annals of hematology.

